# Chair-Stand Exercise Improves Sarcopenia in Rehabilitation Patients after Stroke

**DOI:** 10.3390/nu14030461

**Published:** 2022-01-20

**Authors:** Yoshihiro Yoshimura, Hidetaka Wakabayashi, Fumihiko Nagano, Takahiro Bise, Sayuri Shimazu, Ai Shiraishi, Yoshifumi Kido, Ayaka Matsumoto

**Affiliations:** 1 Center for Sarcopenia and Malnutrition Research, Kumamoto Rehabilitation Hospital, Kumamoto 869-1106, Japan; akro1029@gmail.com (F.N.); asian.dub.foundation00@gmail.com (T.B.); shimazu@kumareha.jp (S.S.); ai.shiraishi0913@gmail.com (A.S.); kidonii921@yahoo.co.jp (Y.K.); ayk1224mtmt@gmail.com (A.M.); 2 Department of Rehabilitation Medicine, Tokyo Women’s Medical University Hospital, Tokyo 162-8666, Japan; noventurenoglory@gmail.com

**Keywords:** activities of daily living, hand strength, muscle, skeletal, resistance exercise, frail elderly

## Abstract

Currently, there is a lack of evidence to show that exercise therapy improves sarcopenia in older patients in clinical practice. We therefore conducted a retrospective cohort study to clarify the effects of chair-stand exercise on improving sarcopenia among patients diagnosed with sarcopenia undergoing convalescent rehabilitation after stroke. According to the latest Asian criteria, sarcopenia was diagnosed when both skeletal muscle mass index (SMI) and handgrip strength (HGS) were low. Patients were asked to perform a repeated chair-stand exercise as whole-body resistance training, in addition to the rehabilitation program. Outcomes included sarcopenia rates, SMI, HGS, and physical function at hospital discharge. Multivariate analyses were used to examine whether the frequency of daily chair-stand exercise was independently associated with the outcomes after adjustment for potential confounders. After enrollment, 302 patients with sarcopenia (mean age: 78.6 years; 46.4% male) were analyzed. Overall, sarcopenia prevalence decreased by 21.9%, from 100% at admission to 78.1% at discharge. Multivariate analyses showed that the frequency of the exercise was significantly associated with the presence of sarcopenia (odds ratio: 0.986, *p* = 0.010), SMI (β = 0.181, *p* < 0.001), and HGS (β = 0.101, *p* = 0.032) at discharge, respectively. The chair-standing exercise was effective in improving sarcopenia in these patients.

## 1. Introduction

Sarcopenia is commonly found in older adults undergoing rehabilitation, and its treatment is mainly based on exercise and nutrition therapy. The prevalence of sarcopenia among patients undergoing rehabilitation is approximately 50% [1,2], which is higher than that of approximately 10% in both genders among older adults in the community [3,4]. The global incidence of sarcopenia is estimated to be about 10%, and the prevalence of sarcopenia is higher in non-Asians than in Asians [5,6,7]. Furthermore, sarcopenia is associated with poor prognosis, including reduced recovery in physical function, dysphagia, incontinence, prolonged hospital stay, and lower rate of home discharge, in these patients [8,9,10,11]. The total cost of hospitalization in patients with sarcopenia is estimated at $40.4 billion, with an average cost per patient of $260 [12]; therefore, early diagnosis of sarcopenia and timely treatment are necessary to maximize improvements in functional recovery.

Exercise and nutritional therapy are the mainstays of treatment for sarcopenia. Currently available guidelines recommend resistance exercise as exercise therapy, and high protein intake and supplementation with essential amino acids as nutritional therapy; however, there is a low level of evidence for each of these therapies [13,14,15]. Furthermore, branched-chain amino acids and leucine are effective in improving muscle-related indices in rehabilitation patients [16].

Stroke is a leading cause of death in many countries, and as a direct consequence, many survivors experience persistent difficulties in activities of daily living (ADL) [17]. Indeed, more than two-thirds of stroke survivors undergo rehabilitation after hospitalization [18]. Further, approximately 50% of stroke patients undergoing rehabilitation have sarcopenia, and sarcopenia interferes with the ability to perform ADL after stroke [19,20,21]; therefore, it is important to combat sarcopenia with exercise and nutritional therapy during stroke rehabilitation [22].

However, there is a lack of evidence to show that exercise therapy improves sarcopenia in older patients undergoing rehabilitation. A recently published systematic umbrella review recommended resistance and combined exercise to improve muscle mass, muscle strength, and physical function in community-dwelling older adults, with moderate-to-high quality of evidence [23]. Multimodal training includes a combination of resistance exercise, aerobic training, walking, and balance training. Although rehabilitation itself has positive effects on preventing muscle weakness and immobility-related complications in hospitalized rehabilitation patients [24], there is little evidence for a specific type of exercise therapy that can improve sarcopenia in these patients. Among exercise therapies, chair-stand exercise, which involves repetitive low-intensity, slow movements, can improve ADL in stroke and dialysis patients [25,26]. The exercise is theoretically expected to improve sarcopenia in addition to conventional rehabilitation programs; however, evidence is lacking at present.

Therefore, in this retrospective cohort study, we aimed to elucidate the effects of chair-stand exercise on improving sarcopenia among stroke patients diagnosed with sarcopenia who were undergoing convalescent rehabilitation.

## 2. Materials and Methods

### 2.1. Participants and Setting

A retrospective cohort study was conducted to analyze stroke patients admitted between 2016 and 2020 in a community-based rehabilitation hospital. The hospital has a total of 135 beds, including three convalescent rehabilitation wards (45 beds each).

All patients admitted to the convalescent rehabilitation wards and diagnosed with sarcopenia were included in the study. Of these, patients who were given permission by their physicians to perform the chair-stand exercise described below were included. Exclusion criteria included patients who refused to give consent to participate, had missing data, had a pacemaker, or had impaired consciousness on the Japan Coma Scale level of 3 digits. The study observation period was the hospitalization period (from the date of admission to the date of discharge).

### 2.2. Convalescent Rehabilitation Program

The rehabilitation program (up to 3 h per day) was tailored to the physical functions and disabilities of each patient [27]. For example, physical therapy included facilitation of paralyzed limb, range of motion training, basic movement training, gait training, resistance training, and ADL training [10].

Nutritional management during the hospital stay was carried out individually according to the nutritional and functional status of the patients under the guidance of a registered dietitian and nutrition support team, including the provision of high-energy, high-protein meals and supplements for malnourished patients, and the provision of sufficient protein and calorie restriction for obese patients to maintain muscle mass [28].

Oral management during hospitalization included oral screening, assessment, patient education, counseling, oral and dysphagia rehabilitation, oral care, dentist treatment, and multidisciplinary practice [29].

Medication management during hospitalization was carried out by multidisciplinary teams, including pharmacists. Medication is one of the factors that affect the physical activity and nutritional status of older adults. Polypharmacy and inappropriate medication were corrected after screening, and medications that may affect physical activity and nutritional status were appropriately managed [30].

### 2.3. “Chair-Stand Exercise” as Whole-Body Resistance Training

**Figure 1:**
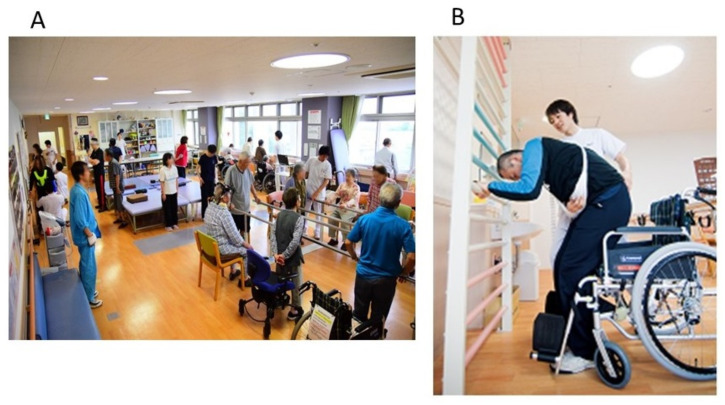
Photos of patients performing chair-stand exercise: (**A**): group chair-stand exercise, (**B**): a post-stroke patient with right hemiplegia standing up from a wheelchair with assistance.

In addition to the individualized structured convalescent rehabilitation program, patients underwent “chair-standing exercise,” a group-based repetition of the task of sit-to-stand from a chair in two sessions per day [16,25]. The chair-stand exercise was an independent program that was clearly distinct from a convalescent rehabilitation program. Using a regular chair, platform, or wheelchair, the seat height was tailored to fit the patient’s physique, approximately 40–50 cm. Patients were able to use the parallel bars and handrails when necessary, and rehabilitation therapists assisted patients who were having difficulty with standing up on their own. Each session lasted 20 min, and the participants were asked to perform a continuous sit-to-stand task up to 120 times at a tempo of about once every 8 s. Patients were asked to count aloud all chair-standing movements, which created a fun and pleasant group atmosphere. On the first day, a few repetitive chair-standing movements were performed, but as muscle strength and endurance improved, the number of repetitions increased day by day. The frequency and degree of increase in chair-standing exercise varied greatly depending on the ability and endurance of each patient (Figure 1).

The chair-stand exercise was performed as part of daily clinical practice, not for research purposes, and the clinical indications were as follows: (1) stable general status; (2) no exacerbation of neurological symptoms; (3) body temperature below 37.5 °C; (4) stable vital signs; and (4) approval from the attending physician to conduct the exercise. There were no requirements for the physical function to engage in the exercises; patients were encouraged to participate in the exercise program, with or without assistance, as long as they could perform at least one chair-standing movement.

In comparison to other whole-body exercises designed for the older population, such as squats, lunges, and balance balls, this exercise is slower and less intense (20–30% of the maximum number of repetitions per repetition), making it safe for older adults with impaired physical function and effective in increasing muscle mass and strength [25]. Moreover, since this movement is closely related to ADL movements, it can be expected to improve the patient’s physical independence. In the current study, we examined the effect of repeated chair-stand exercise on improvement in sarcopenia-related outcomes among patients diagnosed with sarcopenia at admission after stroke.

### 2.4. Data Collection

Basic patient information was collected on admission, including age, gender, type of stroke, body mass index, nutritional status by Mini Nutritional Assessment-Short Form [31], dysphagia or swallowing status by Food Intake Level Scale [32], comorbidities by Charlson Comorbidity Index (CCI) [33], pre-stroke ADL by the modified Rankin Scale [34], and days from onset of stroke to admission. Based on the Brunnstrom Recovery Stages (BRS) [35], the presence of paralysis and the localization and stage of paralysis were collected. The total number of drugs prescribed on admission was recorded from the patient’s medical record.

Within 72 h of admission, skeletal muscle mass index (SMI) by bioimpedance analysis (BIA), handgrip strength (HGS), and functional independence measure (FIM) scores for physical function (FIM-motor) and cognitive function were assessed [36]. BIA measurements (InBody S10; InBody, Tokyo, Japan) were performed according to standard protocols. HGS was measured by a Smedley hand dynamometer (TTM, Tokyo, Japan) with the patient in a standing or sitting position (depending on ability) with the non-dominant hand (or non-paralyzed hand in the case of hemiplegia) and the arm extended horizontally, and the highest of the three measurements was recorded [37]. Energy and protein intakes were estimated by dietitians and nurses by visually determining the ratio of actual intake to the amount provided to the patients. The intake of three meals each for breakfast, lunch, and dinner (nine meals in total) during the first three days after hospitalization was recorded, and the mean value of each meal divided by three was used as the daily intake. The total units of rehabilitation provided during the hospitalization (daily units, 1 unit = 20 min) were recorded based on the medical record.

### 2.5. Sarcopenia Definition

Sarcopenia was diagnosed based on the Asian Working Group for Sarcopenia 2019 criteria and cutoff values if both low SMI and low HGS were present [38]. SMI was calculated from skeletal muscle mass divided by height (in square meters). The cutoff value for SMI was <7.0 kg/m^2^ for men and <5.7 kg/m^2^ for women, and the cutoff value for HGS was <28 kg for men and <18 kg for women, respectively.

### 2.6. Outcome Measures

The main outcome was the presence of sarcopenia at hospital discharge. In the current study, because the patients were diagnosed with sarcopenia at admission, it was determined that the chair-stand exercise would be effective in improving sarcopenia if the number of patients with sarcopenia at discharge decreased. Other outcomes included muscle-related and rehabilitation-related variables. The former included SMI and HGS at discharge, while the latter included FIM-motor at discharge, length of hospital stay (LOS), and home discharge rate.

### 2.7. Sample Size Calculation

In the current study, the target patients were limited to those with sarcopenia. When the median frequency of the chair-stand exercise throughout the study period was used as the cutoff to divide the patients into two groups (a high-frequency exercise group and a low-frequency exercise group), the high- and low-frequency groups were independent samples, and there was one control group per case. Since there was a lack of evidence on the sarcopenia improvement rate, we assumed that the effect of chair-stand exercise on sarcopenia improvement is 10% (with a true sarcopenia probability of 0.90 at hospital discharge) from previous studies on therapeutic interventions for sarcopenia [23,39,40]. Assuming that the effect of chair-stand exercise on sarcopenia is 10%, in this study, with a statistical power of 0.9 and an alpha error of 0.05, we found that a sample size of 98 participants in each group was necessary to reject the null hypothesis.

### 2.8. Ethics Approval

This study was approved by our hospital’s Institutional Review Board (Approval ID: 181-211122). Since this study was conducted retrospectively, no written informed consent was obtained from patients. We instead guaranteed the participants’ right to withdraw from the study through an opt-out procedure. This study was conducted in accordance with the 1964 Declaration of Helsinki and its subsequent amendments, as well as the Ethical Guidelines for Medical and Health Research Involving Human Subjects.

### 2.9. Statistical Analysis

Data are presented as mean (standard deviation (SD)) for parametric data, median and 25–75th percentile (interquartile range (IQR)) for nonparametric data, and numerical values (%) for categorical data. In the bivariate analysis, patients were divided into two groups, one with a low frequency of chair-stand exercise and the other with a high frequency of chair-stand exercise, using the median frequency of chair-stand exercise as a cutoff value. Comparisons between groups were performed by *t*-test, Mann–Whitney U test, and chi-square test.

Multivariate logistic regression analysis was used to examine whether the frequency of chair-stand exercise was independently associated with the presence of sarcopenia at hospital discharge and discharge to home. Multivariate linear regression analysis was used to examine whether the frequency of chair-stand exercise was independently associated with SMI, HGS, FIM-motor score at hospital discharge, and LOS. The covariates selected for bias adjustment were age, gender, LOS, and baseline values of BRS-lower limb, FIM-motor, FIM-cognitive, HGS, SMI, CCI, protein intake, and total rehabilitation therapy (units/day). All of these have been reported to be clinically relevant to rehabilitation outcomes [8,41]. Multicollinearity was evaluated using the variance inflation factor (VIF); if the VIF value was less than 3, it was considered that there was no multicollinearity. A *p*-value of <0.05 was considered statistically significant. Data analysis was performed with IBM SPSS, version 21 (IBM, Armonk, NY, USA).

## 3. Results

**Figure 2:**
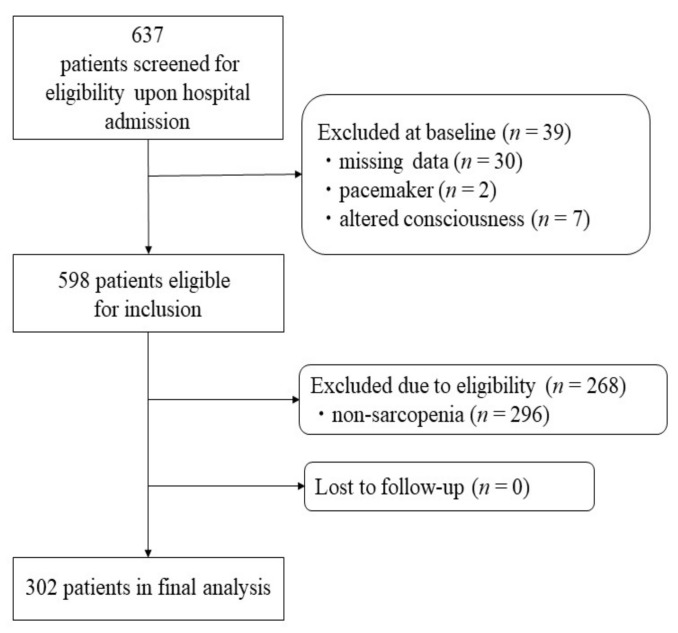
Flowchart of screening, inclusion criteria, and follow-up of participating patients.

A total of 637 stroke patients were hospitalized during the study period. Of these, those with missing data (*n* = 30), pacemaker implantation (*n* = 2), or altered consciousness (*n* = 7) were excluded, resulting in 598 patients screened for eligibility. Of these, 302 patients (50.5%) were diagnosed with sarcopenia at the time of admission, and these were finally included in the study (Figure 2).

The baseline characteristics of the subjects enrolled are summarized in Table 1. The mean (SD) age of the patients was 78.6 (±10.5) years. Forty-six percent of the patients were male. The types of stroke were cerebral infarction (*n* = 196; 64.9%), cerebral hemorrhage (*n* = 94; 31.1%), and subarachnoid hemorrhage (*n* = 12; 4.0%). The median daily frequency (IQR) of chair-stand exercise was 77 (64–97). The median HGS and SMI were 12.6 (5.5–17.7) and 5.1 (4.6–6.0), respectively. The median (IQR) FIM-total, FIM-motor, and FIM-cognitive scores were 44 (26–78), 26 (14–57), and 16 (9–123), respectively, suggesting that many patients had physical dependence at baseline. Bivariate analysis showed that patients with low frequency (exercise frequency < 77 per day) had lower HGS than those with high frequency.

Table 2 shows the results of the univariate analysis of outcomes for the low and high-frequency exercise groups. Overall, the prevalence of sarcopenia decreased by 21.9%, from 100% at admission to 78.1% at discharge. Compared to the low-frequency exercise group, the high-frequency exercise group had a greater increase in SMI (0.4 (0.1, 0.8) vs. 0.1 (−0.1, 0.3), *p <* 0.001) and a greater increase in HGS (3.2 (0.1, 7.5) vs. 1.1 (0.0, 4.5), *p <* 0.001). The prevalence of sarcopenia was reduced by 32.4% in the high-frequency group (median: 98 times/day) and by 11.7% in the low-frequency group (median: 65 times/day) (*p* < 0.001). Compared to patients in the high-frequency exercise group, patients in the low-frequency exercise group had lower SMI (5.1 (4.7–5.9) vs. 5.8 (5.0–6.7), *p* < 0.001) and HGS (13.8 (9.7–19.0) vs. 18.4 (13.0–24.1), *p* < 0.001) at discharge and were more likely to have sarcopenia. There was a strong tendency to have sarcopenia (88.3% vs. 67.6, *p* < 0.001). There was no significant difference in the FIM-motor score at discharge, LOS, and home discharge rate between the two groups.

Table 3 and Table 4 show the results of multivariate analysis for sarcopenia, SMI, and HGS at hospital discharge. There was no multicollinearity between the variables used for adjustment. According to the results of multivariate logistic regression analysis, the frequency of chair-stand exercise showed an independent negative association with sarcopenia at hospital discharge (odds ratio: 0.986, *p* = 0.010). Multivariate linear regression analysis showed that the frequency of chair-stand exercise had independent and positive correlations with SMI (β = 0.181, *p* = < 0.001) and HGS (β = 0.101, *p* = 0.032) at hospital discharge.

Table 5 shows the results of multivariate analysis for FIM-motor score, LOS, and home discharge at the time of hospital discharge. We found no multicollinearity among the variables. According to multivariate linear regression analysis, the chair-stand exercise was independently associated with FIM-movement score at hospital discharge (β = 0.158, *p* = 0.047). There was no significant association of the exercise with LOS and home discharge rate.

## 4. Discussion

The purpose of this study was to investigate the effect of chair-stand exercise on improving sarcopenia in stroke patients diagnosed with sarcopenia undergoing rehabilitation. As a result, two new findings were obtained: (1) chair-stand exercise was positively associated with sarcopenia improvement, and (2) chair-stand exercise was positively associated with ADL improvement.

Chair-stand exercise was positively associated with sarcopenia improvement in patients diagnosed with sarcopenia after stroke. To the best of our knowledge, this is the first cohort study to evaluate the beneficial effects of a specific exercise program on recovery from sarcopenia in a rehabilitation setting. Indeed, in the present study, patients who performed the chair-stand exercise more frequently had a greater reduction in sarcopenia rates. Considering the fact that sarcopenia is found in a high frequency of approximately 50% of rehabilitation patients [1,42] and that sarcopenia has negative impacts on rehabilitation outcomes, whole-body resistance exercise such as the chair-stand exercise in addition to conventional rehabilitation program may improve sarcopenia and maximize the improvement of rehabilitation outcomes such as physical function. Further high-quality studies are needed to examine the effect of specific exercise therapies other than the chair-stand exercise on improving sarcopenia.

The chair-stand exercise was positively associated with ADL improvement. The results indicate that a sufficient number of repetitions of chair stand exercises, in addition to participation in a convalescent rehabilitation program for up to three hours a day, is effective in improving ADL in these patients. It is assumed that repeated chair-standing exercise with a low-intensity resistance training component may promote improvement in ADLs through sarcopenia improvement. In addition, the chair-stand exercise itself may assist in improving the performance of ADL tasks. This exercise involves standing up from a seated position in a chair and enhances the quality of life of post-stroke patients with physical disabilities [43]. Furthermore, this exercise does not require any specialized skills, equipment, or facilities, can be carried out at low cost, and can be carried out by individuals or groups of various sizes even in a hospital rehabilitation environment [25].

Rehabilitation nutrition is important for hospitalized patients undergoing rehabilitation after stroke. Our findings suggest that there is an urgent need to identify sarcopenia at an early stage (upon admission) and initiate whole-body resistance exercises such as chair-stand exercises in order to promote improvement in sarcopenia and physical function in hospitalized patients undergoing rehabilitation. Furthermore, since the mainstay of treatment for sarcopenia includes nutritional therapy [13], early nutritional assessment and timely nutritional supplementation are also necessary to make these exercises multifaceted, especially in patients with sarcopenia. Rehabilitation nutrition is a concept that combines both rehabilitation and nutrition care management [44,45] and can further improve functional outcomes in these patients.

This study had some limitations. First of all, this study was conducted in a single community-based rehabilitation hospital in Japan, which may limit the generalizability of the findings. In the future, multicenter studies are needed to verify whether similar results can be obtained in diverse populations. Secondly, physical function was not used to diagnose sarcopenia. This was because we judged that it was inappropriate to use physical function assessment for sarcopenia diagnosis due to physical disabilities caused by stroke itself. This may affect the accuracy of sarcopenia diagnosis. Third, because of the retrospective study design, we were unable to obtain detailed information on whether the quality and quantity of rehabilitation and nutrition therapy provided during hospitalization affected the results. In the future, high-quality, prospective, and interventional studies that adjust for these confounding factors are needed.

## 5. Conclusions

Chair-stand exercise is positively associated with improvement in sarcopenia and ADL among stroke patients diagnosed with sarcopenia undergoing rehabilitation. In stroke rehabilitation, in order to further improve sarcopenia and ADL, whole-body exercises such as chair-stand exercise should be incorporated in addition to conventional rehabilitation programs.

## Figures and Tables

**Table 1:** Baseline participant characteristics and comparison of low and high-frequency chair-stand exercise between groups.

	Total (N = 302)	Low Freq. Chair-Stand ex. (N = 154)	High Freq. Chair-Stand ex. (N = 148)	*p* Value
Age, years	78.6 (10.5)	78.5 (11.6)	78.6 (9.2)	0.916
Sex, male, *n* (%)	140 (46.4)	64 (41.6)	76 (51.4)	0.106
Stroke type, *n* (%)				
Cerebral infarction	196 (64.9)	97 (63.0)	99 (66.9)	0.547
Cerebral hemorrhage	94 (31.1)	52 (33.8)	42 (28.4)	0.323
Subarachnoid hemorrhage	12 (4.0)	5 (3.2)	7 (4.7)	0.567
Stroke history, *n* (%)	96 (31.8)	43 (27.9)	53 (35.8)	0.174
Premorbid mRS, score	1 (0–2)	1 (0–3)	1 (0–2)	0.165
Onset-admission days	14 (10–22)	14 (10–21)	15 (10–26)	0.065
Paralysis, *n* (%)				
Right/Left/Both	148 (49.0)/98 (32.5)/22 (7.3)	77 (50.0)/48 (31.2)/11 (7.1)	71 (48.0)/50 (33.8)/11 (7.4)	0.731
BRS, score				
Upper limb/Hand-finger/Lower limb	5 (2–6)/5 (2–6)/5 (2–6)	4 (2–5)/5 (2–6)/5 (2–6)	4 (2–6)/5 (2–6)/5 (2–6)	0.312
FIM, score				
-Total	44 (26–78)	42 (25–78)	49 (26–80)	0.245
-Motor	26 (14–57)	26 (13–56)	27 (14–56)	0.609
-Cognitive	16 (9–23)	15 (8–23)	17 (10–26)	0.098
FILS, score	7 (2–9)	7 (2–8)	7 (2–9)	0.111
CCI, score	3 (2–4)	3 (2–4)	3 (2–4)	0.143
Nutritional status				
MNA-SF, score	5 (3–8)	5 (3–8)	6 (3–8)	0.694
BMI, kg/m^2^	21.2 (18.7–22.7)	21.2 (18.7–22.9)	21.0 (18.9–22.7)	0.656
Energy intake, kcal/kg/day	27.9 (24.1–32.5)	27.6 (23.8–32.6)	28.1 (24.2–32.4)	0.609
Protein intake, g/kg/day	1.1 (0.9–1.3)	1.1 (0.9–1.3)	1.1 (0.9–1.3)	0.915
Muscle-related variables				
HGS, kg	12.6 (5.5–17.7)	10.30 (1.0–15.2)	14.0 (6.7–18.9)	0.001
SMI, kg/m^2^	5.1 (4.6–6.0)	5.0 (4.7–5.9)	5.4 (4.6–6.2)	0.072
Laboratory data				
Alb, g/dL	3.4 (0.5)	3.42 (0.49)	3.49 (0.51)	0.237
CRP, g/dL	1.2 (2.2)	1.44 (2.64)	1.17 (1.72)	0.092
Hb, mg/dL	12.9 (1.5)	12.65 (1.58)	12.96 (1.53)	0.124
Number of drugs	5 (3–8)	5 (3–8)	5 (3–8)	0.593
Rehabilitation therapy, units/day	8.2 (7.2–8.5)	8.2 (7.5–8.4)	8.1 (6.9–8.5)	0.400
Chair-stand exercise, frequency/day	77 (64–97)	65 (52–76)	98 (88–111)	-

BMI, body mass index; BRS, Brunnstrom Recovery Stage; CCI, Charlson’s Comorbidity Index; FILS, Food Intake Level Scale; FIM, Functional Independence Measure; FM, fat mass; FMI, fat mass index; HGS, handgrip strength; MNA-SF, Mini Nutritional Assessment-Short Form; mRS, modified Rankin Scale; SAH, subarachnoid hemorrhage; SMI, skeletal muscle mass index.

**Table 2:** Bivariate analysis of outcomes in the low-frequency chair-stand exercise group and the high-frequency chair-stand exercise group.

	Total (N = 302)	Low Freq. Chair-Stand ex. (N = 154)	High Freq. Chair-Stand ex. (N = 148)	*p* Value
SMI, kg/m^2^				
change (pre-post exercise)	0.2 (0.1–0.5)	0.1 (−0.1–0.3)	0.4 (0.1–0.8)	<0.001
at discharge	5.4 (4.8–6.3)	5.09 (4.71–5.90)	5.83 (5.00–6.70)	<0.001
HGS, kg				
change (pre-post exercise)	2.4 (0.0–6.8)	1.1 (0.0–4.5)	3.2 (0.1–7.5)	<0.001
at discharge	15.5 (11.0–22.4)	13.80 (9.72–18.95)	18.40 (12.95–24.05)	<0.001
Sarcopenia at discharge, number	236 (78.1)	136 (88.3)	100 (67.6)	<0.001
FIM-motor at discharge*, score*	61 (37–83)	55 (36–79)	72 (40–83)	0.052
Length of hospital stay*, days*	117 (73–142)	118 (79–131)	107 (71–128)	0.141
Home discharge, *number*	154 (51.0)	70 (45.5)	84 (56.8)	0.051

FIM, Functional Independence Measure; HGS, handgrip strength; SMI, skeletal muscle mass index.

**Table 3:** Multivariate analysis for SMI, handgrip strength, and sarcopenia at hospital discharge in men.

	SMI at Discharge			HGS at Discharge			Sarcopenia at Discharge	
	β	B (95% CI)	*p* Value	Β	B (95% CI)	*p* Value	OR (95% CI)	*p* Value
Age	−0.060	−0.005 (−0.014, 0.005)	0.315	−0.130	−0.099 (−0.200, 0.002)	0.054	1.091 (1.021, 1.165)	0.010
LOS	0.110	0.002 (−0.001, 0.005)	0.118	0.085	0.018 (−0.015, 0.050)	0.278	0.961 (0.934, 0.989)	0.007
FIM-motor	−0.057	−0.002 (−0.011, 0.006)	0.579	−0.052	−0.022 (−0.114, 0.071)	0.646	0.906 (0.842, 0.976)	0.009
FIM-cognitive	−0.036	−0.004 (−0.021, 0.013)	0.652	0.114	0.117 (−0.066, 0.300)	0.207	0.957 (0.863, 1.061)	0.402
CCI	−0.005	−0.003 (−0.063, 0.058)	0.930	−0.045	−0.207 (−0.846, 0.431)	0.521	0.638 (0.404, 1.010)	0.055
Rehabilitation	0.026	0.009 (−0.028, 0.046)	0.617	0.055	0.182 (−0.207, 0.572)	0.355	1.073 (0.857, 1.343)	0.540
BRS-lower limb	0.153	0.071 (0.002, 0.141)	0.045	−0.084	−0.368 (−1.103, 0.366)	0.323	0.734 (0.419, 1.283)	0.278
Protein intake	−0.091	−0.358 (−0.785, 0.070)	0.101	0.053	1.966 (−2.555, 6.486)	0.391	0.573 (0.014, 2.191)	0.771
HGS at admission	0.006	0.001 (−0.014, 0.015)	0.927	0.520	0.517 (0.361, 0.672)	<0.001	1.045 (0.948, 1.152)	0.374
SMI at admission	0.770	0.933 (0.792, 1.074)	<0.001	0.338	3.860 (2.368, 5.353)	<0.001	0.012 (0.001, 0.116)	<0.001
Chair-stand exercise, freq./day	0.270	0.008 (0.005, 0.012)	<0.001	0.131	0.039 (0.002, 0.075)	0.039	0.968 (0.945, 0.992)	0.010
R^2^	0.772			0.751			0.431	

**Table 4:** Multivariate analysis for SMI, handgrip strength, and sarcopenia at hospital discharge in women.

	SMI at Discharge			HGS at Discharge			Sarcopenia at Discharge	
	β	B (95% CI)	*p* Value	Β	B (95% CI)	*p* Value	OR (95% CI)	*p* Value
Age	−0.174	−0.016 (−0.026, −0.007)	0.001	−0.131	−0.125 (−0.270, 0.020)	0.089	1.035 (0.979, 1.094)	0.229
LOS	0.044	0.001 (−0.002, 0.003)	0.500	0.078	0.015 (−0.021, 0.051)	0.417	0.993 (0.978, 1.009)	0.379
FIM-motor	−0.084	−0.003 (−0.012, 0.005)	0.412	−0.296	−0.122 (0.243, −0.001)	0.048	1.030 (0.987, 1.075)	0.178
FIM-cognitive	0.027	0.003 (−0.013, 0.018)	0.722	0.087	0.095 (−0.138, 0.327)	0.423	0.960 (0.878, 1.051)	0.377
CCI	0.075	0.047 (−0.014, 0.108)	0.131	−0.054	−0.343 (−1.253, 0.566)	0.457	1.149 (0.788, 1.674)	0.470
Rehabilitation	−0.033	−0.023 (−0.102, 0.057)	0.574	0.102	0.718 (−0.464, 1.900)	0.232	0.725 (0.413, 1.272)	0.262
BRS-lower limb	0.028	0.012 (−0.048, 0.072)	0.689	0.039	0.172 (−0.716, 1.061)	0.702	0.931 (0.671, 1.291)	0.666
Protein intake	−0.097	−0.252 (−0.529, 0.025)	0.074	−0.083	−2.196 (−6.305, 1.913)	0.293	1.472 (0.642, 2.783)	0.148
HGS at admission	0.098	0.014 (−0.007, 0.036)	0.188	0.629	0.940 (0.620, 1.259)	<0.001	0.873 (0.779, 0.978)	0.019
SMI at admission	0.750	0.956 (0.825, 1.086)	<0.001	−0.014	−0.188 (−2.127, 1.752)	0.849	0.180 (0.066, 0.490)	0.001
Chair-stand exercise, freq./day	0.206	0.004 (0.002, 0.006)	<0.001	0.067	0.028 (0.016, 0.045)	0.037	0.992 (0.979, 0.998)	0.012
R^2^	0.712			0.689			0.398	

BRS, Brunnstrom Recovery Stage; CCI, Charlson’s Comorbidity Index; FIM, Functional Independence Measure; HGS, handgrip strength; LOS, length of hospital stay; SMI, skeletal muscle mass index.

**Table 5:** Multivariate analyses for FIM-motor at discharge, length of hospital stay, and home discharge.

	FIM-Motor at Discharge			Length of Hospital Stay			Home Discharge	
	β	B (95% CI)	*p* Value	Β	B (95% CI)	*p* Value	OR (95% CI)	*p* Value
Age	−0.051	−0.125 (−0.314, 0.063)	0.192	−0.195	−0.831 (−1.228, −0.433)	<0.001	0.996 (0.963, 1.031)	0.834
Sex (male)	−0.308	−15.955 (−21.167, −10.743)	<0.001	0.098	8.832 (−2.421, 20.084)	0.124	0.890 (0.769, 0.1522)	0.101
Los	0.097	0.056 (0.003, 0.109)	0.040	-	-	-	1.008 (0.998, 1.017)	0.129
FIM-motor	0.370	0.443 (0.280, 0.605)	<0.001	−0.424	−0.878 (−1.214, −0.541)	<0.001	1.075 (1.042, 1.109)	<0.001
FIM-cognitive	0.097	0.297 (−0.021, 0.614)	0.067	0.066	0.350 (−0.338, 1.038)	0.317	1.020 (0.969, 1.075)	0.446
CCI	−0.050	−0.778 (−1.959, 0.403)	0.196	0.109	2.957 (0.420, 5.494)	0.023	0.894 (0.730, 1.093)	0.274
Rehabilitation	0.069	0.870 (−0.056, 1.797)	0.066	−0.063	−1.395 (−3.398, 0.608)	0.172	1.411 (1.065, 1.870)	0.017
BRS-lower limb	0.199	2.548 (1.219, 3.877)	<0.001	−0.169	−3.748 (−6.597, −0.900)	0.010	1.094 (0.878, 1.362)	0.424
Protein intake	−0.061	−5.257 (−11.947, 1.434)	0.123	0.031	4.608 (−9.887, 19.102)	0.532	1.019 (0.982, 1.121)	0.122
HGS at admission	0.251	0.802 (0.469, 1.134)	<0.001	−0.074	−0.412 (−1.132, 0.308)	0.261	1.065 (1.007, 1.125)	0.027
SMI at admission	0.269	6.951 (4.234, 9.667)	<0.001	−0.104	−4.664 (−10.528, 1.200)	0.119	2.046 (1.246, 3.361)	0.005
Chair-stand exercise, freq./day	0.158	0.041 (0.010, 0.098)	0.047	−0.065	−0.080 (−0.191, 0.031)	0.159	1.005 (0.995, 1.016)	0.288
R^2^	0.617			0.598			0.554	

BRS, Brunnstrom Recovery Stage; CCI, Charlson’s Comorbidity Index; FIM, Functional Independence Measure; HGS, handgrip strength; LOS, length of hospital stay; SMI, skeletal muscle mass index.

## Data Availability

The data are not publicly available owing to opt-out restrictions. Data sharing is not applicable.
